# Acrylamide adsorption by *Enterococcus durans* and *Enterococcus faecalis*: *In vitro* optimization, simulated digestive system and binding mechanism

**DOI:** 10.3389/fmicb.2022.925174

**Published:** 2022-11-08

**Authors:** Amal S. Albedwawi, Reem Al Sakkaf, Tareq M. Osaili, Ahmed Yusuf, Anas Al Nabulsi, Shao-Quan Liu, Giovanni Palmisano, Mutamed M. Ayyash

**Affiliations:** ^1^Department of Food Science, College of Agriculture and Veterinary Medicine, United Arab Emirates University (UAEU), Al Ain, United Arab Emirates; ^2^Department of Chemical Engineering, Center for Membrane and Advanced Water Technology (CMAT), Research and Innovation on CO2 and Hydrogen (RICH), Khalifa University of Science and Technology, Abu Dhabi, United Arab Emirates; ^3^Department Clinical Nutrition and Dietetics, University of Sharjah, Sharjah, United Arab Emirates; ^4^Department of Nutrition and Food Technology, Faculty of Agriculture, Jordan University of Science and Technology, Irbid, Jordan; ^5^Department of Food Science and Technology, Faculty of Science, National University of Singapore, Singapore, Singapore

**Keywords:** acrylamide, LAB, TEM, SEM-EDS, FTIR, Box–Behnken design, the reduction mechanism

## Abstract

Acrylamide is an unsaturated amide that forms in heated, starchy food products. This study was conducted to (1) examine the ability of 38 LAB to remove acrylamide; (2) optimize acrylamide removal of selected LAB under various conditions (pH, temperature, time and salt) using the Box–Behnken design (BBD); (3) the behavior of the selected LAB under the simulated gastrointestinal conditions; and (4) investigate the mechanism of adsorption. Out of the 38 LAB, *Enterococcus durans* and *Enterococcus faecalis* had the highest results in removing acrylamide, with 33 and 30% removal, respectively. Those two LAB were further examined for their binding abilities under optimized conditions of pH (4.5–6.5), temperature (32°C - 42°C), time (14–22 h), and NaCl (0–3% w/v) using BBD. pH was the main factor influenced the acrylamide removal compared to other factors. *E. durans* and *E. faecalis* exhibited acrylamide removal of 44 and 53%, respectively, after the *in vitro* digestion. Zeta potential results indicated that the changes in the charges were not the main cause of acrylamide removal. Transmission electron microscopes (TEM) results indicated that the cell walls of the bacteria increased when cultured in media supplemented with acrylamide.

## Introduction

Acrylamide (A.A., 2-propenamide, C_3_H_5_NO, Mr. = 71.09) is an unsaturated amide that can form in heated food products rich in carbohydrates. In 2002, the Swedish National Food Authority and Stockholm University announced that acrylamide could form in food heated above 120°C (Perera et al., 2021). Baking, frying, or roasting food rich in reducing sugar and the amino acid “asparagine” in low-moisture conditions can cause acrylamide formation (Mottram et al., 2002; Claeys et al., 2005). Acrylamide is characterized as a colorless, odorless, crystalline, water-soluble amide that thermally decomposes to CO_2_, CO, NO_2_, and NH_3_. Acrylamide is absorbed by the organs of humans and animals after ingestion and increases the risk of cancer (Capuano and Fogliano, 2011). In addition, acrylamide can cause neurological damage in humans. Therefore, in 1994, The International Agency of Research on Cancer (IARC) classified acrylamide in the “2A Group” as a potential carcinogen (probable human carcinogen; International Agency for Research on Cancer, 1994).

Since its detection in food in 2002, acrylamide has captured the attention of scientists, food safety authorities, and governments to take imminent actions and measures to prevent or mitigate its formation (Mottram et al., 2002). Many food products were examined to analyze acrylamide levels and assess the risk to human health (Food and Agricultural Organization of the United Nations/World Health Organization (FAO/WHO), 2011; European Food Safety Authority (EFSA), 2015). High quantities of acrylamide were found in products like potato chips, bread, and coffee (Shao et al., 2021). The European Food Safety Authority has established guidelines on the maximum allowable limits of acrylamide in food, stating the indicative acrylamide levels’ indicative values (European Food Safety Authority (EFSA), 2015). Additionally, the World Health Organization (WHO) has specified the maximum limit of acrylamide levels in drinking water to be 0.5 mg/kg (Ahn et al., 2002).

Several approaches were considered to mitigate or eliminate acrylamide in food. Pre-processing of raw food materials reduced its precursors, preventing its formation. An example of this approach is controlling the storage conditions of raw potatoes. The cooler the storage conditions, the more reducing sugar will form, leading to higher acrylamide formation (Viklund et al., 2008; Khorshidian et al., 2020). Processing the food and changing its cooking parameters, such as pH, additives, moisture content, shortening cooking time, and fermenting it, can also impact acrylamide formation (Anese et al., 2010; Khorshidian et al., 2020). However, these approaches can have an undesirable effect on the sensory properties of the final food products (Shen et al., 2019b).

Lactic acid bacteria (LAB) were also tested for their ability to bind acrylamide after its formation in food. (Anese et al., 2010; Serrano-Niño et al., 2014). LAB are recognized Generally Recognized as Safe (GRAS), as stated by the U.S. Food and Drug Administration. LAB are, as potential probiotic, living microorganisms with health-related benefits for the host if given sufficient quantities (Kerry et al., 2018). LAB belong to a wide class of Gram-positive, facultatively anaerobic, immotile microorganisms, acid-resistant cocci, and bacilli, which can ferment sugars into lactic acid (Shoukat, 2020; Bangar et al., 2021). LAB may prevent microbial spoilage, which can then expand the shelf-life of food (Bangar et al., 2021). Additionally, LAB can remove heavy metals from food, such as cadmium, lead, aluminum, and other toxins, such as aflatoxin, acrylamide, and zearalenone through absorption, adsorption, and/or degradation (Adunphatcharaphon et al., 2021; Bangar et al., 2021; George et al., 2021).

After the publication of research by Serrano-Niño et al. (2014), several studies were conducted to understand the mechanism of acrylamide removal by lactobacillus strains. It was reported that the cell wall had a major role in the adsorption of the toxin (Serrano-Niño et al., 2015; Wang et al., 2015; Sangsila et al., 2016; Zhang et al., 2017). However, it has been reported that additional effort is needed to study the surface characteristics of LAB strains, their cell wall composition, and the adsorption ability of A.A. (Zhang et al., 2017; Shen et al., 2019b). For example, adding *Lb. bulgaricus* and *Lb. rhamnosus* to fired potatoes and fermented bread, respectively, removed 85% of the dietary A.A. (Albedwawi et al., 2021). With that being said, and with additional research needed to understand the behavior of LAB and its ability to remove A.A., this study aimed to investigate: (1) the ability of 38 newly isolated LAB to remove A.A.; (2) the behavior of the selected two LAB strains under optimized conditions of pH levels, incubation temperature, time and NaCl levels using BBD; (3) the removal ability of selected LAB isolates in an *in-vitro* simulated digestion system using the INFOGEST2.0 model and (4) characterization of the cell wall of the chosen strains, using scanning electron microscopy in combination with energy-dispersive X-ray spectroscopy (SEM-EDS), zeta potential, transmission electron microscopy (TEM), and Fourier transforms infrared spectroscopy (FTIR) to interpret the mechanism of adsorption.

## Materials and methods

### Bacterial strains

Thirty-eight strains of LAB strain that our laboratory had already isolated from food products (Abushelaibi et al., 2017; Ayyash et al., 2018; Alkalbani et al., 2019) were examined for their ability to mitigate the production of acrylamide. The isolates were maintained at −20°C in a stock of 50% glycerol. The classification of these strains was as the following genera: (1) *Enterococcus*; (2) *Pediococcus*; and (3) *Streptococcus*. A loopful of the bacterial stock was inoculated into 10 ml of a broth of de Man, Rogosa, and Sharpe (MRS) (LAB-M, Neogen Culture Media, Heywood, United Kingdom) and then incubated at 37°C for 20 h to activate the isolates.

### Preparation of stock and working solutions of acrylamide

We prepared an acrylamide stock solution by dissolving 50 mg of powdered acrylamide (purity >99%, Sigma Chemical Co., St. Louis, MO, USA) in a 50-ml volumetric flask of deionized water, giving a concentration of 1 mg/ml. This was used in the screening stage to prepare two working solutions, diluting the stock solution to 50 μg/ml and 100 μg/ml in 10 ml of MRS broth. These working concentrations were selected based on the AA concentrations used in various food system reported in the previous studies (Albedwawi et al., 2021).

### Acrylamide binding assay – Preliminary screening for media components and bacterial cultures

An aliquot of 1% of activated culture was inoculated into two samples of 10 ml of sterilized MRS broth supplemented with acrylamide, one at 50 μg/ml and the other at 100 μg/ml. The samples were then incubated at 37°C for 20 h. Subsequently, a pour-plate method was used to perform bacterial enumeration with MRS agar, and anaerobic incubation was at 37°C for 20 h. In preparation for acrylamide analysis by LC–MS–MS, bacterial cells were removed from the samples by centrifugation (10,000 x g, 10 min). Acrylamide analysis was conducted on the supernatants collected, with each sample being analyzed in duplicate. Each group of samples was matched with controls of MRS broth, MRS with bacteria, and MRS spiked with acrylamide without bacteria (Albedwawi et al., 2022).

### Optimization of acrylamide removal using BBD

BBD was employed for the optimization of the four variables, each at three levels: pH of 4.5, 5.5, and 6.5, using 1.0 mol/l HCl or 1.0 mol/l NaOH; the temperature of 32°C, 37°C, and 42°C, using anaerobic incubators; incubation period of 14, 18 and 22 h; and NaCl of 0%, 1.5 and 3% w/v. Experiment design and statistical analysis were performed using Minitab v.21. Twenty-seven experimental runs with three repetitive central points were used to investigate the four independent variables. Two different LAB strains were investigated under aerobic and anaerobic conditions, with each experiment conducted in triplicate, as shown in Tables 1, 2. The polynomial equation is given below:

**Table 1:** Box–Behnken experimental design with coded variables and responses of acrylamide removal (%) by *E. durans* and *E. faecalis* under anaerobic conditions.

Runs	Temperature (°C) (X1)	pH (X2)	Time (h) (X3)	NaCl (g/100 ml) (X4)	(%) AA removal	
					*E. durans*	*E. faecalis*
1	42	6.5	18	0.0	39.1	44.7
2	42	5.5	18	1.5	2.5	47.3
3	42	5.5	14	1.5	4.5	41.2
4	37	6.5	18	1.5	35.4	49.2
5	37	5.5	22	0.0	37.4	45.4
6	32	5.5	22	1.5	33.5	49.4
7	37	4.5	14	3.0	35.8	47.5
8	37	4.5	18	1.5	32.8	44.1
9	42	5.5	18	1.5	13.2	49.0
10	37	5.5	18	0.0	33.3	45.7
11	37	5.5	14	1.5	25.8	50.6
12	32	6.5	18	3.0	26.9	51.2
13	37	6.5	22	1.5	26.4	53.8
14	37	6.5	14	1.5	24.3	49.6
15	37	5.5	18	0.0	25.7	45.1
16	32	5.5	18	0.0	33.0	41.3
17	37	6.5	18	0.0	26.2	52.8
18	37	5.5	14	3.0	37.4	47.1
19	42	4.5	18	3.0	17.2	50.1
20	32	5.5	14	1.5	34.5	48.5
21	37	4.5	18	1.5	45.1	50.5
22	37	5.5	22	1.5	37.4	37.8
23	32	4.5	18	3.0	25.8	47.3
24	37	5.5	18	3.0	40.0	43.7
25	37	4.5	22	1.5	37.3	44.6
26	32	5.5	18	1.5	37.1	48.6
27	42	5.5	22	1.5	2.7	45.3

**Table 2:** Analysis of variance for *E. durans* and *E. faecalis.*

Source	*E. durans*			*E. faecalis*	
	DF	*F*-value	*p*-value	*F*-value	*p*-value
Model	14	1.47	0.257	0.89	0.587
Linear	4	2.11	0.142	0.99	0.449
Temp	1	7.90	0.016	1.66	0.222
pH	1	0.01	0.931	1.99	0.183
Time	1	0.29	0.598	0.14	0.714
Salt	1	0.00	0.945	0.00	0.981
Square	4	1.61	0.234	1.03	0.433
Temp*Temperature	1	3.65	0.080	0.03	0.855
pH*pH	1	0.14	0.719	0.26	0.617
Time*Time	1	0.61	0.450	0.72	0.412
Salt*Salt	1	0.00	0.962	2.60	0.133
2-Way Interaction	6	0.28	0.933	0.74	0.628
Temperature*pH	1	0.29	0.598	2.62	0.132
Temperature*Time	1	0.00	0.970	0.18	0.680
Temperature*Salt	1	0.02	0.900	0.64	0.438
pH*Time	1	0.14	0.712	0.24	0.633
pH*Salt	1	0.01	0.936	1.79	0.205
Time*Salt	1	0.84	0.376	0.46	0.511
Error	12				
Lack-of-Fit	9	2.30	0.266	2.26	0.272
Pure Error	3				
Total	26				
Model Summary					
S		10.3231		3.78545	
*R*-sq		63.09%		50.95%	
*R*-sq(adj)		20.03%		0.00%	
*R*-sq(pred)		0.00%		0.00%	


$$Y=\beta_{0}+\sum \beta_{i}X_{i}+\sum \beta_{ii}X_{i}^{2}+\sum \beta_{ij}X_{i}X_{j}$$

Where Y is the predicted acrylamide concentration or OD600 nm, and Xi and Xj are the independent variables. The variables studied were pH, temperature, time, and NaCl. βₒ is the regression coefficient of the model, and βi, βii, and βij are the linear, quadratic, and interaction coefficients, respectively. The relationship between the independent variables and the responses was investigated by constructing two-dimensional response surface plots. The ANOVA results provided corresponding *p*-values to evaluate the significance (Albedwawi et al., 2022).

Optical density at 600 nm was used to measure the microbial population using a spectrophotometer and 24-well plates, using an Epoch 2 Microplate Spectrophotometer from BioTeck, Santa Clara, California, United States. After determining the bacterial populations, 1.5-ml tubes were used to collect the samples and centrifuge at 10,000 × *g* for 10 min. The supernatants collected were further analyzed for acrylamide using Agilent Technologies 6,495 Triple-Quad LC–MS–MS (Santa Clara, California, USA).

### *In-vitro* digestion by INFOGEST2.0 model

The *in-vitro* gastrointestinal INFOGEST2.0 protocol (Brodkorb et al., 2019) was applied to selected LAB strain samples (*E. durans* and *E. faecalis*). A 1-ml aliquot of the bacterial pellet suspension and aliquot of AA were subjected to *in-vitro* digestion named INFOGEST2.0 model. The sequence of (1) oral phase (amylase 75 U/ml, simulated salivary fluid (SSF), 0.3 M CaCl_2_, 2 min, 37°C), (2) gastric phase (pepsin 2000 U/ml, simulated gastric juice (SGF), pH 3.0, 0.3 M CaCl_2_, 120 min, 37°C) omitting the rabbit gastric extract, followed by (3) intestinal phase (pancreatin 100 U/ml, bile 10 mmol/l, simulated intestinal juice (SIF) pH 7.0, 0.3 M CaCl_2_, 120 min, 37°C) was followed. The samples were continuously shaken at 120 rpm during whole *in vitro* digestion process (4 h and 2 min). During INFOGEST2.0 treatment, samples were collected for the bacterial count and acrylamide analysis. The bacterial count was measured by serial dilution immediately after taking the samples. For acrylamide analysis, the samples were frozen under −20°C for later analysis as described in section 2.6.

### Quantification of acrylamide by LC–MS–MS

To determine the amount of acrylamide in the aqueous fraction, the Agilent 1,290 Infinity LC system was used. It was equipped with the MS/MS detector (Agilent, Santa Clara, CA, USA), Column Hypercarb C_18_ (2.1 × 100 mm, 5.0 μm, Thermo Scientific, Waltham, MA, USA). The 1% acetic acid mobile phase in deionized water with a 0.2 ml/min flow rate was employed. An injection volume of 20 μl and a column temperature of 35°C were applied. For quantification, an external acrylamide standard curve of 0, 5, 10, 25, 50, 75, 100, 125 and 150 𝜇g/ml was constructed.

### Understanding the mechanism of acrylamide binding by LAB

#### Preparation of samples and binding assay

The two LAB strains were activated twice in MRS broth for 24 h at 37°C. To obtain 10^9^ CFU/ml of the second sub-culture, an aliquot of the activated cultures from the second sub-culture was added at 1% v/v to 10 ml of fresh MRS broth containing 10 μg/ml acrylamide. This was incubated at 37°C with 0% NaCl and pH 6.5 for 18 h. Following incubation, samples were centrifuged at 5,000 x *g* for 10 min at 4°C. Supernatants were extracted, and bacterial cells were collected in 0.1 M, pH 7.0 phosphate buffer in 1.5-ml tubes and retained under −20°C until analysis.

#### Estimation of zeta potential

The LAB cells’ zeta potential was measured using a micro electrophoretic apparatus, Zeta Plus (Zetasizer Nano Z.S. 90, Malvern Instruments Ltd., Worcestershire, U.K.). The experiment was conducted at ~23°C (room temperature), with the pH adjusted using 0.1 M NaOH and 0.1 M HCl (Shen et al., 2019b).

#### Fourier transform infrared spectroscopy analysis

FTIR analysis was carried out to determine the functional groups and putative binding sites potentially impacting acrylamide adsorption. This analysis involved attenuated total reflectance (ATR)-FTIR spectroscopy, for which we used a Spectrum Two I.R., combined with a Universal ATR (UATR) unit (Perkin-Elmer Inc., Norwalk, CT, United States). Freeze-dried bacterial cell samples were directly positioned on a Diamond/ZnSe crystal plate (Perkin-Elmer). The IR spectral range was 4,000–400 cm^−1^ (Lin et al., 2011; Ge et al., 2017; Shen et al., 2019b).

#### Scanning electron microscopy coupled with energy-dispersive X-ray spectroscopy

The morphology and elementary composition of bacterial cells were examined by SEM-EDS. Acrylamide was added to the samples, and bacterial cells were tested and fixed with 2.5% (v/v) glutaraldehyde containing 1% osmium tetroxide. The radius, height, and elemental composition of *E. durans* and *E. faecalis* were evaluated by Quanta 250 ESEM. (Ge et al., 2017; Shen et al., 2019a). From each sample, a 5-μL amount was placed on a piece of aluminum foil attached to a stainless-steel stub with carbon tape. This was allowed to dry before loading the SEM device using a stub holder.

#### Transmission electron microscopy measurement

LAB cells were characterized by employing a Tecnai G2 TEM operating at 200 kV. The prepared samples were cut into 50–60 nm sections of thickness using an Ultramicrotome (UC6, LEICA, Wetzlar, Germany; Shen et al., 2019b).

### Statistical analysis

The Minitab v.21 (Minitab Ltd., Coventry, United Kingdom) was used to calculate the mean values and standard deviations of the results obtained by screening the samples. BBD was employed, with the responses analyzed using the Minitab v.21.

## Results

### Screening of acrylamide removal by LAB

**Figure 1:**
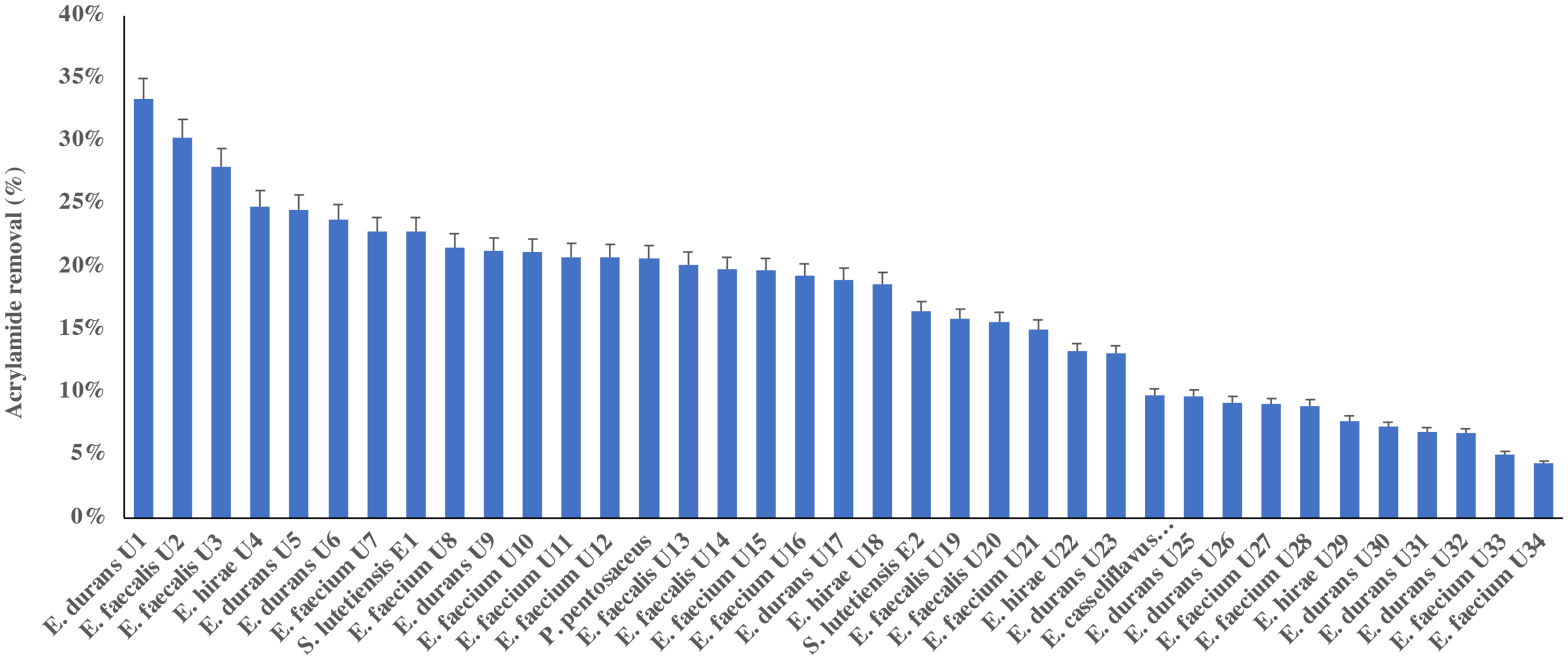
Acrylamide removal (%) by 38 lactic acid bacteria isolates. The bar value of triplicates. Error bars present the standard deviation.

Figure 1 presents the results of screening 38 LAB for their ability to remove acrylamide. The conventional method of screening LAB and their ability to bind acrylamide showed positive results for all strains (Figure 1). The removal percentages ranged from 4 to 33%. The strains *E. durans* (accession no. KY795325) (33%) and *E. faecalis* U2 (accession no. MF067501) (30%) had the highest % acrylamide removal.

It has been reported that acrylamide removal by microorganisms is species- and strain-dependent (Albedwawi et al., 2021). Unlike many other genera of LAB, *Enterococcus* has not yet been approved as Generally Recognized as Safe by the U.S. Food and Drug Administration (Nascimento et al., 2019). *E. durans* strains are described as useful for both health- and food-related applications to produce short-chain fatty acids. Both *E. faecalis* and *E. durans* have been isolated from the raw milk of cows, goats, and sheep and are used to ferment cheese and other products (Dapkevicius et al., 2021). In this study, *E. faecalis* and *E. durans* strains were selected for further analysis for acrylamide removal based on their screening results.

### Optimization of acrylamide removal

**Figure 2:**
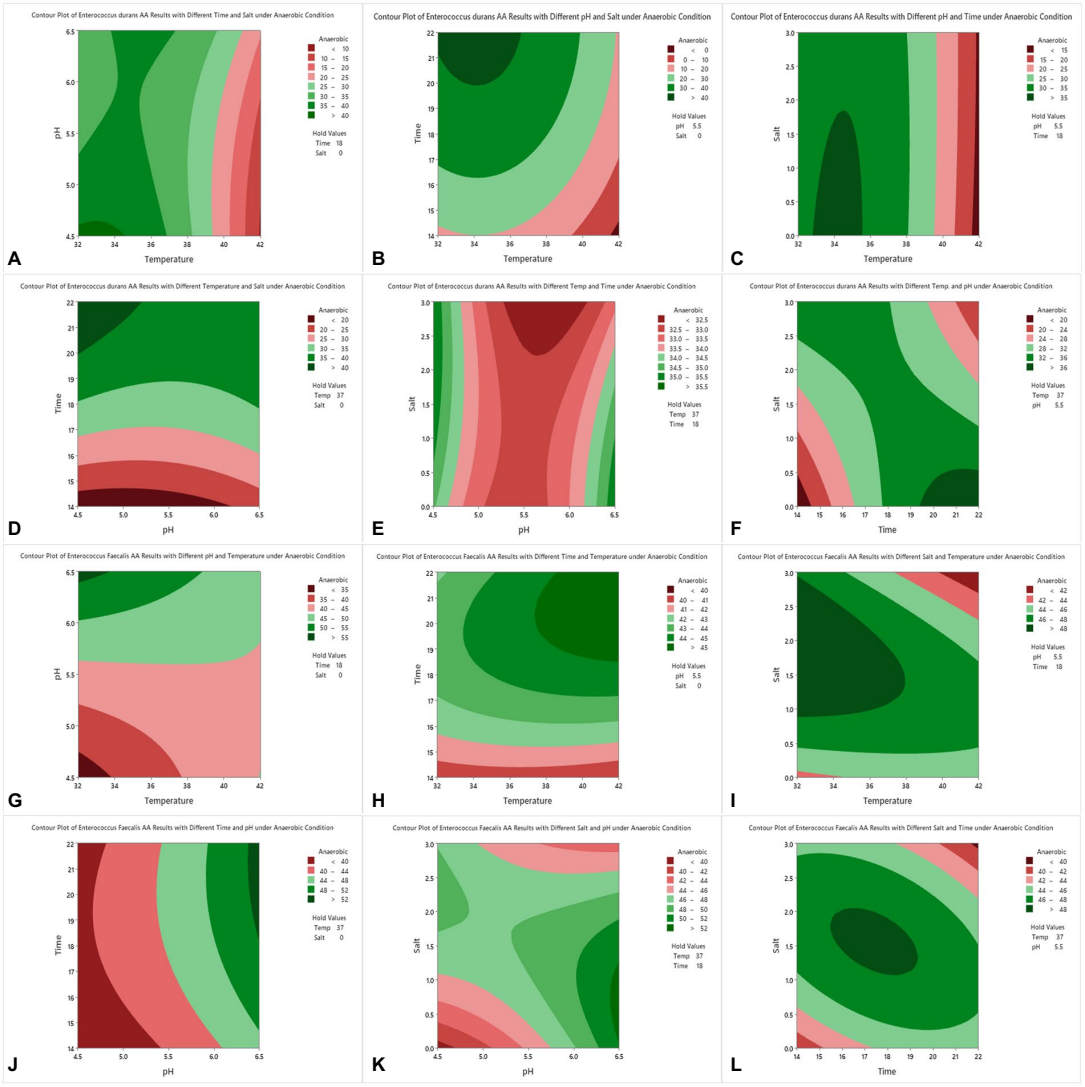
Contour plots of acrylamide removal in anaerobic conditions for *E. durans* **(A–F)** and *E. faecalis* **(G–L)** under controlled conditions of incubation time of 14–22 h, salt (NaCl) of 0.0–3.0%, incubation temperature of 32–42°C and pH of 4.5–6.5.


Table 1 presents the number of runs and the optimized conditions of temperature, pH, time, and NaCl using BBD with the responses of acrylamide levels by percentages for both *E. durans* and *E. faecalis* under anaerobic conditions. Table 2 presents the analysis of variance for both strains. Figures 2A–L are the contour plots of responses of acrylamide vs. the optimized factors after running the experiments using BBD under anaerobic conditions. After running the 27 experiments for both *E. durans* and *E. faecalis*, as presented in Table 1, and running an analysis of variance (ANOVA), two regression equations resulted that describe the actual relationships between the responses and the independent variables for both strains:

$$\begin{array}{cc} \begin{array}{l} \mathrm{Acrylamide removal}\;\\ \mathrm{by}\;E.durans \end{array} & \begin{array}{c} =-330+20.1\;X_{1}-53\;X_{2}+16.6X_{3}\\ +26.1X_{4}-0.375X_{1}{}^{2}+2.27X_{2}{}^{2}\\ -0.288X_{3}{}^{2}-0.15X_{4}{}^{2}+1.04X_{1}X_{2}\\ -0.010X_{1}X_{3}+0.13X_{1}X_{4}-0.55X_{2}X_{4}\\ -0.49X_{2}X_{4}+1.56X_{3}X_{4} \end{array} \end{array}$$

$$\begin{array}{cc} \begin{array}{l} \mathrm{Acrylamide removal}\;\\ \mathrm{by}\;E.faecalis \end{array} & \begin{array}{c} =-204+6.64X_{1}+30.9X_{2}+1.73X_{3}\\ +40.2X_{4}-0.0134X_{1}{}^{2}-0.115X_{3}{}^{2}\\ -1.78X_{4}{}^{2}-1.135X_{1}X_{2}+0.0400X_{1}X_{3}\\ -0.302X_{1}X_{4}+0.264X_{2}X_{3}-2.93X_{2}X_{4}\\ -0.421X_{3}X_{4} \end{array} \end{array}$$

Table 2 presents the analysis of variance (ANOVA) of both *E. durans* and *E. faecalis*. Both strains had statistically insignificant results for both *F*-value and *p*-value: *F*-value = 1.47 and *p*-value was insignificant (*p* > 0.257) for *E. durans* and *F*-value = 0.89 and *p*-value of (*p* > 0.587) for *E. faecalis*. The coefficient of determination values *R*^2^ was 0.631 for all models of *E. durans*, suggesting that the developed models have the goodness of fit that could explain >63% of the total variation. The adjusted *R*^2^ was 20.03% for *E. durans*. *E. faecalis* had lower results, with a goodness of fit that could explain up to 50.95% of the total variation. The *p*-values for the lack of fit for the models were not significant for both strains: *p* > 0.266, *p* > 0.272 for *E. durans* and *E. faecalis*, respectively.

For *E. durans*, Figures 2A–F shows the effect of salt, incubation temperature, pH, and incubation time on the bacteria’s ability to reduce acrylamide under different conditions. Acrylamide was at its lowest level at NaCl of 0.0 with incubation temperature of 32–37°C, pH 4.5–5.5, and incubation time of 18–22 h, but these relationships are statistically insignificant (*p* > 0.05), except for the relation with temperature alone (*p* < 0.016). The level of acrylamide was reduced by more than 40% under those conditions, as shown in Figures 2A,B,D. Figures 2D,F show that incubating the bacteria for 14 h had the least acrylamide removal. Thus, we conclude that the longer the incubation time, the higher the binding results. For *E. faecalis*, there were no significant relationships between pH, incubation temperature, salt, and incubation time, nor their interactions and acrylamide levels, as shown in Table 2. In Figure 2G *E. faecalis* indicates higher acrylamide removal than *E. durans* under the same conditions as in Figure 2A. *E. faecalis* performed better under pH 6.5, temperature 32–34°C, incubation time 18 h, and salt 0.0.

By analyzing Figures 2A–L and observing the interaction between the factors and acrylamide removal by both strains of LAB, we note that *E. durans* acted better in a warm, acidic environment that lacks NaCl, unlike *E. faecalis*, which can bind high levels of acrylamide in warm salty surroundings with pH of 6.5. The longer the strains were incubated with the acrylamide, the higher the binding results.

### Acrylamide removal under *in-vitro* digestion

**Figure 3:**
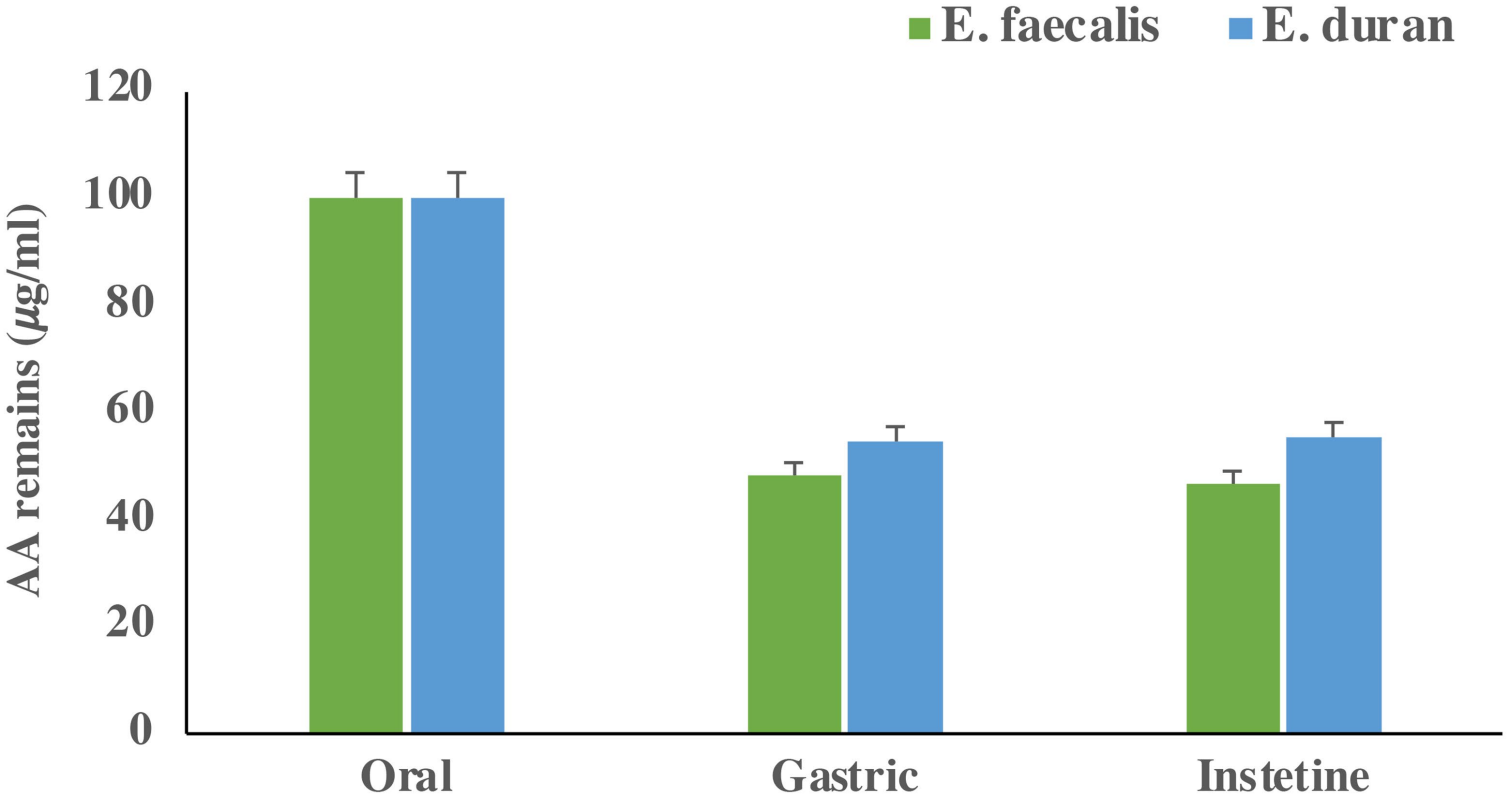
Acrylamide concentration (𝜇g/mL) remain in GIT fluid after each step. Bars present means of triplicates and error bar present standard deviation.

The remaining acrylamide in the gastrointestinal fluids are presented in Figure 3. The percentages of acrylamide removal were 53.34 and 44.67% for *E. faecalis* and *E. durans*, respectively. This indicates that acrylamide removal is species-dependent under the simulated gastrointestinal system. The results indicate that LAB can remove acrylamide formed during the cooking processes in food during *in vitro* digestion. The effect of the matrix on acrylamide removal during *in vitro* digestion was also investigated. Our results showed that the media matrix (the *in vitro* digestion solutions) had a minor effect (< 1.1%) on acrylamide removal.

### Mechanisms of acrylamide removal

#### Estimation of zeta potential of bacterial cells

The average zeta potentials of *E. durans* and *E. faecalis* suspensions were − 11.37 and − 26.27 mV, respectively. Both strains showed poor coagulation results and high adsorption rates, as presented in Figure 1. The zeta potential results of both strains greatly varied, despite their high adsorption results, suggesting that the zeta potential of *E. durans* and *E. faecalis* might not relate to their binding abilities.

#### Fourier transform infrared spectroscopy analysis

**Figure 4:**
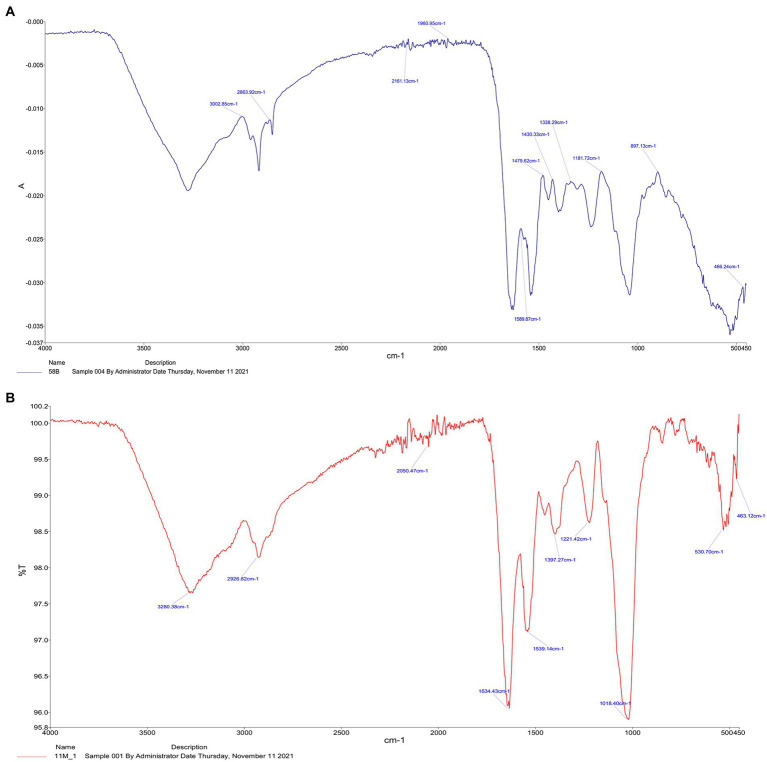
FTIR spectra of *E. durans* **(A)** and *E. faecalis* **(B)**.

The FTIR spectra of *E. durans* and *E. faecalis* are presented in Figures 4A,B. The two strains showed different peaks, reflected by the differences in the functional groups C-O, C=O, and N-H, which might lead to variations in adsorption capacities. FTIR spectrum of for *E. durans* and *E. faecalis* showed distinct peaks at the following bands. The strong absorption band was around 1800 cm-1 which may arise due to the stretching vibrations of C=O. The medium absorption bands located at 2860 and 2,900 cm −1 corresponds to C-O stretch. The band at 3002 cm − 1 indicates presence of amine group (Figures 4A,B). In the comparison with the FTIR spectrum of the acrylamide, the major shift was observed in the carbonyl, hydroxyl and amino group of protein, which may be due to the composition of the cell walls of the *E. durans* and *E. faecalis*.

Polysaccharides and proteins in the cell wall have been reported to be related in the adsorption of toxins by both yeast and bacteria (Zoghi et al., 2014). Both results of FTIR and EDS in the present paper supported Shen et al. (2019b) and Haskard et al. (2000) who stated that carbohydrates and protein components of LAB cell walls played a major role in adsorption of patulin and aflatoxin B1, where the current paper added that cell wall can also bind acrylamide (Haskard et al., 2000; Shen et al., 2019b). The peptidoglycan layer of the cell wall for different stains contains CeO, OH and NH functional groups as major components. Additionally, the peptidoglycan contains amino acids as part of its structure (Niderkorn et al., 2009). Those studies suggest that the cell wall composition can bind toxins and this paper suggests that it can also bind or react with acrylamide.

#### Scanning electron microscopy coupled with energy-dispersive X-ray spectroscopy

**Figure 5:**
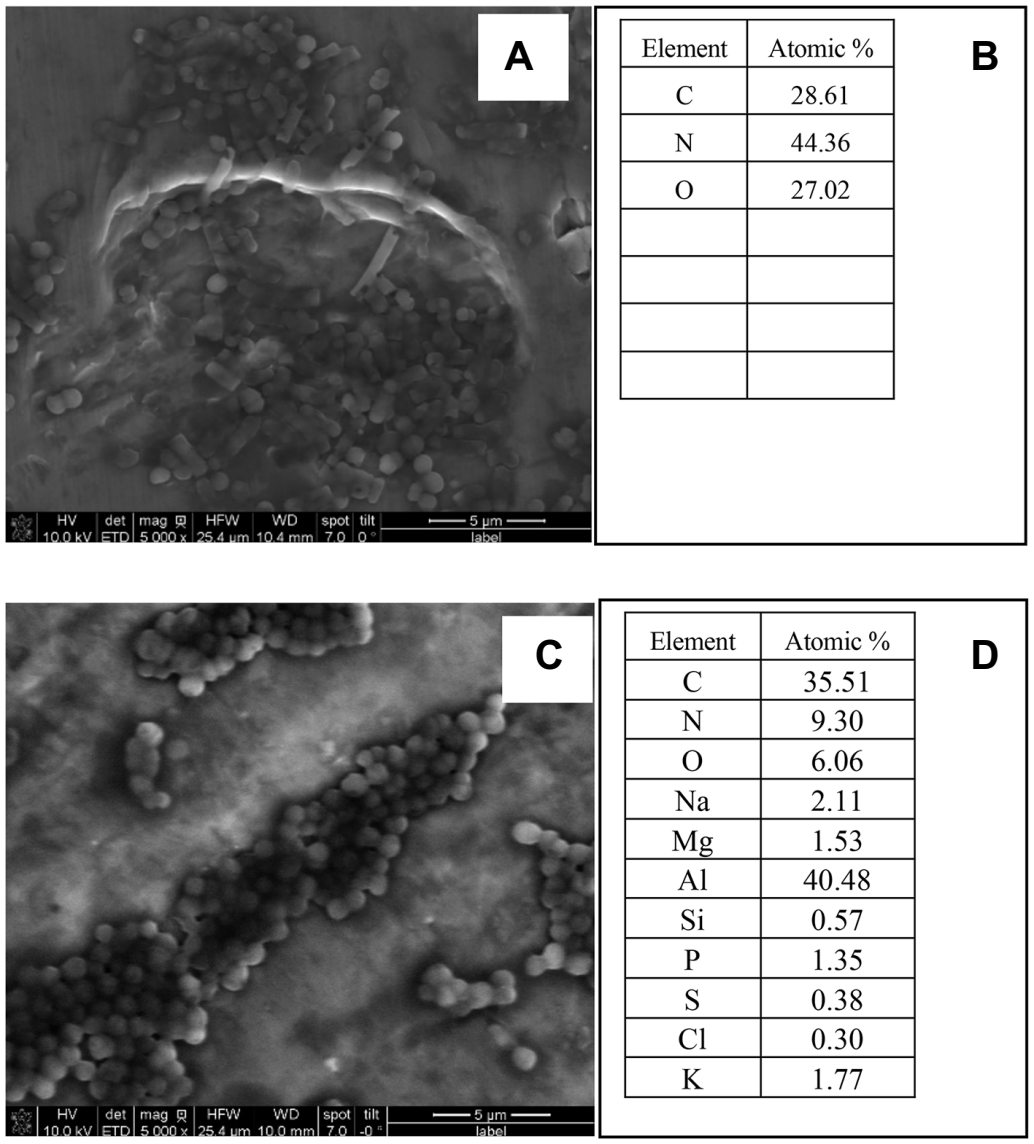
SEM-EDS images and elements of *E. durans* **(A,B)** and *E. faecalis* **(C,D)** and the peaks of the EDS of the main elements.

Figures 5A–D presents the elements of *E. durans* and *E. faecalis*. Figure 5B shows that the most dominant elements in *E. durans* were C, N, and O, whereas *E. faecalis* had Al, C, N, O, Mg, and P as the most dominant, as presented in atomic percentages in Figure 5D. *E. durans* and *E. faecalis* had significant differences in the composition of elements. Interestingly, *E. faecalis* contained more elements than *E. durans*, but both had components of oxygen (O), nitrogen (N), and carbon (C), which might explain their ability to adsorb acrylamide.

#### Transmission electron microscopy measurement

**Figure 6:**
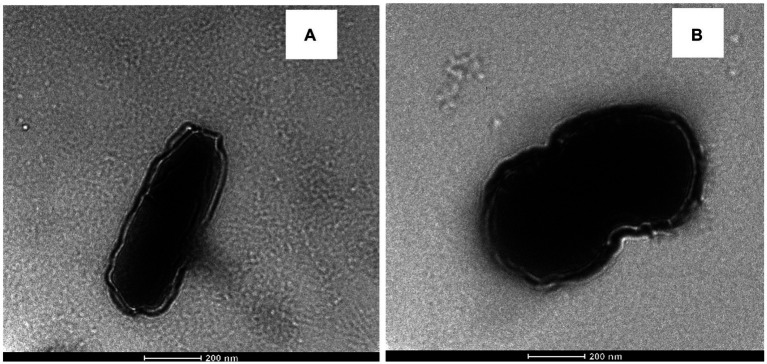
TEM of **(A)** *E. durans* and **(B)** *E. faecalis* showing the shape of the cells and the thickness of the cell walls with exposure to acrylamide.

TEM images of *E. durans* and *E. faecalis* are displayed in Figures 6A,B. Based on the visual observations, the TEM images showed that the cell wall structure changed after introducing acrylamide to the bacteria. The cell wall of both strains became wrinkled, longer, and thicker, which changed its original shape, which might explain the higher level of acrylamide adsorption.

## Discussion

After the announcement that acrylamide posed a risk to human health and could be formed in cooked starchy food, researchers studied several approaches to its removal and elimination. In comparison to many approaches that have been suggested, such as changing raw materials, adding new ingredients to recipes, or changing the processing method, LAB presented positive results in removing acrylamide without affecting the sensory characteristics of food (Elmore et al., 2015; Xu and An, 2016; Nematollahi et al., 2021).

The present study found that *E. durans* and *E. faecalis* had the ability to reduce the levels of acrylamide in MRS medium by up to 33 and 30%, respectively. In a previous study, *Streptococcus lutetiensis* and *Lactiplantibacillus plantarum* removed up to 39 and 26% of acrylamide, respectively (Albedwawi et al., 2022). Additionally, Serrano-Niño et al. (2014) reported that *Limosilactobacillus reuteri* 14,171 and *Lacticaseibacillus casei* Shirota were the most efficient binders, with removal percentages of 24.01 and 24.95, respectively, after an incubation time of 12 h *in vitro* (Serrano-Niño et al., 2014). Serrano-Niño et al. (2014) applied the same method used by Hernandez-Mendoza et al. (2009) to analyze acrylamide removal using LAB whereas Hernandez-Mendoza et al. (2009) used *L. casei* to bind aflatoxin B1, with the highest removal of 49.2% by *L. casei* L30 (Hernandez-Mendoza et al., 2009).

The present study reports that the LAB removal conditions can be optimized using BBD for *E. durans* and *E. faecalis*, increasing the binding from 33 to 45.1% and 30 to 53.8%, respectively. The optimal removal conditions for *E. durans* were: (1) NaCl 0.0; (2) incubation temperature 32–34°C; (3) pH 4.5–5.5 and (4) incubation time 18–22 h. Similarly, Serrano-Niño et al. (2014) stated that pH, the concentration of acrylamide, and the type of strains were some of the factors that impacted LAB binding ability and acrylamide removal (Serrano-Niño et al., 2014). They applied BBD to optimize the binding conditions of benzo[a]pyrene for four strains of LAB, where *Lactobacillus acidophilus* had the highest removal under 10 ppm Bbenzo[a]pyrene concentration, pH 5, cell density 10^9^ CFU/ml, and incubation period of 24 h (Yousefi et al., 2021). Both studies showed that optimizing the conditions increased the binding results of LAB.

After introducing LAB *in vitro* into a simulated gastrointestinal system, both *E. durans* and *E. faecalis* had similar removal results under optimized conditions, and both tolerated a low pH environment. *S. lutetiensis* and *L. plantarum* removed by 30 and 40%, respectively, under the same conditions as in a previous study (Albedwawi et al., 2022). Under simulated gastrointestinal conditions, *L. reuteri* and *L. casei* Shirota were tested to remove acrylamide from potato chips. They removed 73 and 39%, respectively, under the stimulated gastrointestinal conditions (Rivas-Jimenez et al., 2016).

The present study supported the results of previous studies, which stated that the C=O, C-O & N-H groups were the primary functional groups involved in the adsorption of acrylamide by LAB according to FITR and SEM-EDS (Ge et al., 2017; Shen et al., 2019b; Albedwawi et al., 2022). Those groups are the key components of the peptidoglycan and the cell wall proteins (Zhang et al., 2017; Shao et al., 2021). Shen et al. (2019b) also reported that increasing cell wall roughness can improve the adsorption capacity of the strain to acrylamide (Shen et al., 2019b; Shao et al., 2021). Petka et al. (2020) supported the previous results and stated that acrylamide had an impact on the viability of *L. plantarum* by decreasing its count and changing its morphology which was also found in the present study by TEM for both enterococcal strains, where the outer layer became wrinkled, increasing its binding area (Petka et al., 2020). It was also reported that *Lactobacillus kefiranofacein* was used to adsorb the mycotoxin patulin in apple juice (Bahati et al., 2021). The bacteria’s cell wall was the main contributor that bound the toxin, and the thicker it was, the higher the adsorption results.

## Conclusion

Acrylamide is a carcinogenic chemical that forms in heated, starchy food. Forty LAB strains have shown different acrylamide-binding abilities, and *E. durans* and *E. faecalis* had the highest capability of acrylamide removal at 33 and 30%, respectively. In a simulated intestinal tract system, both strains removed more than 44–53% of the acrylamide. *E. durans* and *E. faecalis* could bind acrylamide *in vitro* under controlled and simulated gastrointestinal conditions. The suggested removal mechanism supported the previous study on the role of the cell wall and the importance of understanding its structure in removing acrylamide. The functional C=O, C-O, and N-H are the main causes of the adsorption, along with the deformed shape of the cell wall. Cell wall charges were not involved in the removal. This study indicates that LAB might be used, in the future, to remove toxins in food and human intestines because LAB can tolerate different conditions. Further research is needed *in vivo* to test LAB’s ability in binding acrylamide and other toxins in the human gastrointestinal system.

## Data availability statement

The original contributions presented in the study are included in the article/supplementary material, further inquiries can be directed to the corresponding author.

## Author contributions

AA: investigation, writing–original draft, and formal analysis. RA and AY: investigation. TO, AA, and GP: supervision and writing–review and editing. S-QL: writing–review and editing. MA: conceptualization, writing–original draft, funding, writing–review and editing and supervision. All authors contributed to the article and approved the submitted version.

## Funding

This research and APC were funded by United Arab Emirates University (UAEU), Al-Ain, UAE.

## Conflict of interest

The authors declare that the research was conducted in the absence of any commercial or financial relationships that could be construed as a potential conflict of interest.

## Publisher’s note

All claims expressed in this article are solely those of the authors and do not necessarily represent those of their affiliated organizations, or those of the publisher, the editors and the reviewers. Any product that may be evaluated in this article, or claim that may be made by its manufacturer, is not guaranteed or endorsed by the publisher.
